# Trichinellosis dissemination among wild carnivores in the Republic of Kazakhstan: A 10-year study

**DOI:** 10.14202/vetworld.2023.1840-1848

**Published:** 2023-09-14

**Authors:** Orken S. Akibekov, Alfiya S. Syzdykova, Lyudmila A. Lider, Aibek Kh. Zhumalin, Fariza S. Zhagipar, Aissarat M. Gajimuradova, Sergey N. Borovikov, Zhanbolat A. Suranshiyev, Sagandyk A. Ashimov

**Affiliations:** Department of Microbiology and Biotechnology, Faculty of Veterinary and Livestock Technology, Saken Seifullin Kazakh Agrotechnical Research University, 62 Zhenis Avenue, Astana, 010000, Kazakhstan

**Keywords:** distribution, intensity of infection, trichinellosis, wild carnivores

## Abstract

**Background and Aim:**

Trichinellosis is caused by a species of roundworm called *Trichinella* and is an invasive disease causing severe medical, veterinary, and socioeconomic problems worldwide. More than 100 mammalian species are *Trichinella* hosts. Among domestic animals, pigs and dogs are prone to trichinellosis. An essential aspect of controlling the spread of infection is to identify the number and level of infections in wild carnivores in the country. However, the number, habitats, and movements of wild animal *Trichinella* hosts in Kazakhstan have not been reported yet. This study aimed to monitor the wild animal habitat nearby the settlements for tracking the trichinellosis speading among carnivores.

**Materials and Methods:**

Wild carnivorous animals were captured in seven regions of the Republic of Kazakhstan. The carcasses of corsacs, wolves, foxes, wild boars, and badgers were studied. Muscle tissue samples from spontaneously infected wild animals were collected. The digestion method in “GASTROS-2M” was used to isolate *Trichinella* spp. from animal muscles. The species of the parasite was determined by a polymerase chain reaction for 5S spacer of *Trichinella* ribosomal DNA with subsequent sequencing by Senger. Statistical analysis methods were performed for average value in Microsoft Excel 2010.

**Results:**

The results of the research showed that among 155 animals wolves (20.4%) and foxes (26.7%) were the most infected with invasive Trichinella larvae. The invasion intensity was 503.6% in foxes and 289.7% in wolves. However, badgers (164%), wild boars (0%), and corsacs (0%) presented lower invasion levels. Using specific primers, larvae samples were identified as *Trichinella nativa*.

**Conclusion:**

The results of monitoring revealed the spread of trichinosis among wild animals: wolves, foxes, badgers. The Karaganda, Kostanay, Western Kazakhstan, and Akmola regions had the largest distribution of wild animals infected with trichinellosis. In total, 20% of the 155 studied animals were infected. The greatest invasion intensity was typical for wolves, foxes and badgers. It is necessary to monitor the spread of trichinellosis among wild carnivores to control the epidemiological situation and reduce the level of spontaneous infection among animals. Regular monitoring of habitats and carnivores must be conducted within the country and in the border areas.

## Introduction

Determining the role of wild animals in disseminating dangerous pathogens to humans and domestic animals and preserving natural focal zoonoses are crucial. Trichinellosis a helminthic zoonosis, representing a severe epidemic and worldwide epidemiological danger for humans and domestic animals [1–3]. Trichinellosis is a human and mammalian intestinal and tissue helminthiasis caused by *Trichinella* spp. worldwide. The *Trichinella* genus is a complex of ten species and at least three genotypes with an uncertain taxonomic status [4]. However, no morphological differences exist between the two main clades, except for the presence or absence of a collagen capsule (cyst) surrounding the parasite larvae in infected muscles. Each taxon can be identified using polymerase chain reaction (PCR) and enzyme-linked immunosorbent assay performed on larvae and blood serum collected from infected human or animal muscle samples [5].

*Trichinella spiralis* is the most common and widespread encapsulated species affecting humans and domestic animals in temperate regions [6]. *Trichinella nativa* is found in Arctic and subarctic predators [7], and *Trichinella* britovi is found primarily in carnivores and less often in domestic and wild pigs throughout the European continent, northwest Africa, and southwest Asia [8]. *Trichinella murrelli* is found only in carnivores in North American temperate regions [9]. *Trichinella nelsoni* is present in carnivores and less often in wild pigs in eastern and southern Africa [10]. *Trichinella patagoniensis* is found in carnivores in South America [11]. *Trichinella pseudospiralis*, *Trichinella papuae*, and *Trichinella zimbabwensis* make up the unencapsulated clade (i.e., devoid of a cyst in an infected muscle). *Trichinella pseudospiralis* has a cosmopolitan distribution and is the only species capable of infecting mammals and birds. In Australia and Southeast Asia, *T. papuae* circulates among mammals (e.g., wild pigs) and reptiles (saltwater crocodiles and soft-shell turtles). *Trichinella zimbabwensis* affects carnivorous mammals (e.g., lions, leopards, and spotted hyenas), Nile crocodiles, and Nile monitor lizards in sub-Saharan Africa [12].

Humans can become infected with *Trichinella* when eating improperly cooked or raw pork, horse, meat from other domestic animals, or wild animal meat such as bear or wild boar. Some reports mention accidental infection when eating reptile meat, including lizards and turtles. However, no human-to-human parasite transmission has been reported [13, 14]. Nematodes of the genus *Trichinella* persist in the wild cycle, and their epidemiology is closely related to the nutritional behavior of host species. The domestic cycle is auxiliary to the wild cycle and begins when domestic animals are fed which remains of domesticated or wild animals infected with *Trichinella*. The primary *Trichinella* reservoir hosts are carnivores and a few omnivorous species with scavenger behavior (possums, mice). *Trichinella* larvae can survive in the muscle tissues of infected animals for months to several years, depending on the species, and their development continues only if another suitable host ingests them. Infected cells degenerate as the larva grows, followed by calcification, occurring at different rates in different hosts. After the death of the host, the larvae remain viable and contagious in decomposing muscles for several weeks. Humans can considerably influence the wild cycle by promoting or reducing the transmission of these pathogens through unfair utilization of infected carcasses.

Epidemiological information on animal and human *Trichinella* infections is available for 95 countries. In the 21^st^ century, cycles of wild and domestic animals were recorded in 75 and 32 countries, respectively [15]. Approximately 0.58 cases of Trichinellosis per 100,000 population are registered annually in Kazakhstan; however, these indicators are unreliable. These incidents were primarily reported due to undercooked wild animal or dog meat consumption. In 2006–2010, the incidence rate of trichinellosis was 0.1–0.2/100 thousand population in Kazakhstan. Analyzing the trichinellosis incidence in humans showed that the invasion method in most cases was the consumption of meat from stray dogs (37 cases, 63.9%), badger (10 cases, 17.2%), wild boar (10 cases, 17.2%), and wolf (one case, 1.7%) meat [16].

Thus, to this day, monitoring the habitat of animal populations, especially near settlements and on the territories of hunting farms, is relevant for studying trichinellosis infection. During the autumn–winter period, *Trichinella* spp. larvae are concentrated in the carcasses of commercial carnivores in rural settlements. Therefore, studying the level of trichinellosis invasion in the Republic of Kazakhstan is crucial. Tracking migrating animals outside the country may allow for keeping records of infected wild animals and determining the trichinellosis distribution areas. The population should also be informed about the distribution areas of infected animals to prevent spontaneous infection of people and domestic animals.

In each region of Kazakhstan, trichinellosis is characterized by a natural focal character. However, interest in trichinellosis has recently been declining in our region. Scant data on the dynamics of the spread of trichinellosis among predisposed animals are available, making it challenging to organize and perform preventive measures.

Therefore, this study aimed to estimate the prevalence of trichinellosis among wild carnivores, the species diversity of *Trichinella*, and the intensity of animal invasion in seven regions of the Republic of Kazakhstan over 10 years.

## Materials and Methods

### Ethical approval

This study was approved by the Animal Ethics Committee of the Faculty of Veterinary and Livestock Technology, S. Seifullin Kazakh Agrotechnical Research University by protocol no. 1 from 23^rd^ of February 2023. All protocols were performed following the International Guiding Principles for Biomedical Research Involving Animals [17].

### Study period and location

The study was conducted from October 2013 to February 2023 at the scientific and technical base in the Laboratory of Parasitology and the Research Platform of Agricultural Biotechnology, Faculty of Veterinary Medicine and Animal Husbandry Technology, S. Seifullin Kazakh Agrotechnical Research University.

### Animals

Muscle tissue samples of spontaneously infected wild animals were used in the study. Chilled and frozen (−20°C) internal organs of wild canids were obtained by licensed shooting and delivered to the laboratory. Official permission to remove animal species, which is subject to regulation in various regions of the Republic of Kazakhstan, was obtained (order of the Minister of Ecology, Geology, and Natural Resources of the Republic of Kazakhstan dated December 30, 2020, No. 347. Registered with the Ministry of Justice of the Republic of Kazakhstan on December 31, 2020, No. 22000).

### Larvae digestion

*Trichinella* spp. larvae were diagnosed and isolated from animal muscle tissue samples using compressor trichinelloscopy and digestion in artificial gastric juice (IHS), recommended by the FLOUR 4.2.2747-10: “Methods of sanitary and parasitological examination of meat and meat products.” For sample peptolysis, the classical method, in which digestion is carried out without activating the medium for 16–18 h, and the hardware method using a special “GASTROS-2M” equipment for 30 min were performed. Compressors and watch glasses with isolated sediment were microscopically examined using an MBS-100 T microscope (Labex Instrument Limited, China) at 16–32× magnification.

The following indices were used to determine the quantitative and qualitative indicators of invasion and accumulation of *Trichinella* larvae in naturally infected hosts: Invasion intensity (II) and abundance index (AI). The invasion extent was defined as the ratio of the number of infected hosts to the total number of examined animals and expressed as a percentage (%). The II was determined by the number of detected *Trichinella* larvae in 1 g of the calf muscle group (lich./g, ex./g). The AI is the average number of *Trichinella* in all individuals of a specific host species, including uninfected hosts. Muscle tissue attachments were weighed using an electronic scale.

### Species identification

*Trichinella* species typing was performed using molecular genetic analysis. The detected and isolated helminthological material was preserved in a 70% ethanol solution. DNA from each helminthic sample was extracted using the DNA IQ System Kit (Promega, USA) according to the manufacturer’s instructions and used for PCR. Briefly, 20 mL of incubation buffer with DTT and proteinase K were added to the *Trichinella* larvae and incubated at 55°C for 30 min, with intermittent shaking. Then, 40 mL of lysis buffer with DTT and 4 mL of paramagnetic resin were added and incubated at 25°C for 5 min in a thermoblock without vibration, with one shaking step performed in the middle. The tubes were then placed in a magnetic separation stand for 1 min, 100 μL of lysis buffer was added before replacing the tubes, and the resin particles were re-suspended. Samples were washed 4 times using 100 μL of washing buffer. The concentration of the isolated DNA was measured using a NanoDrop 2000 spectrophotometer (Thermo Scientific, Waltham, MA, USA), and the concentrations varied from 132 to 241 ng/mL. Mitochondrial DNA fragments of cytochrome C oxidase subunit I were amplified using five specific species primers directed at the internal transcribed site of ITS1, ITS2, and ESV, which are used to differentiate all currently known *Trichinella* genotypes, including three genotypic isolates of *T. pseudospiralis* [18]. *Trichinella* products were electrophoresed on 2% agarose gel (Sigma-Aldrich, USA) using a BioRad electrophoresis camera at 125 mA and 250 W.

### Sequencing

To amplify the intergenic region of the 5S spacer of *Trichinella* ribosomal DNA, PCR was performed on the VeritiPro Thermal Cycler (Applied Biosystems, Waltham, MA, USA) using the Taq 5X Master Mix (M0285L, New England Biolabs, Ipswich, MA, USA) and previously published primers (5′-GCGAATTCTTGGATCGG AGACGGCCTG-3′; R 5′-GCTCTAGACGAGATGT CGTGTGTTTCAACG-3′) [17].

The obtained amplicons were purified using ExoSAP-IT (GE Healthcare, Chicago, Il, USA) according to the manufacturer’s instructions. A total of 70 ng of the purified product was sequenced in a reaction with 4 pmol PCR primers using a ready-made sequencing Big dye terminator 3.1 kit (Applied Biosystems). All reactions were performed on a SeqStudio sequencing machine Genetic Analyzer System (Applied Biosystems, USA). Polymerase chain reaction products were sequenced in the 3′ and 5′ directions.

### Statistical analysis

All experiments were conducted 3 times, while PCR analysis was performed 2 times. Statistical analysis was performed according to the generally accepted methodology, with the standard average deviation calculated using Microsoft Excel 2010 (Microsoft, USA).

## Results

### Level of *Trichinella* infection in wild animals

**Figure-1:**
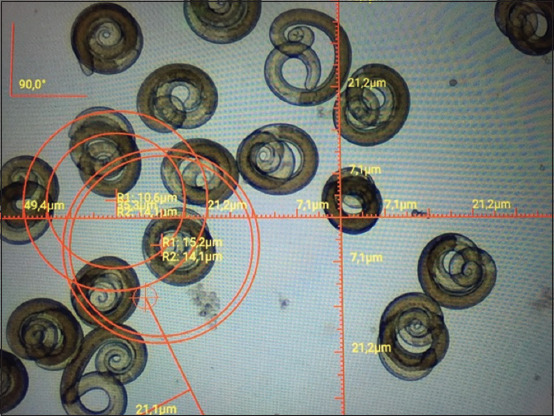
*Trichinella* larvae after digestion in artificial gastric juice in the muscles of a badger from the Kyzylzhar district, Kazakhstan.

During 10 years of research, 155 carcasses of slaughtered animals captured on the territory of seven regions in three parts of the country were examined. From October 2013 to March 2015, the muscles of eight corsacs and three badgers from different regions in the northern region of Kazakhstan (Akmola, North Kazakhstan reg.) were examined. Two badgers were infected with *Trichinella*, as determined using trichinoscopy and digestion in artificial gastric juice. Two badgers were caught in the North Kazakhstan region in the Kyzylzhar district and one in the Akmola region (Korgaldzhinsky district). A total of 70 *Trichinella* larvae per 1 g of muscle tissue were isolated from a badger from the Kyzylzhar district (Figure-1).

**Figure-2:**
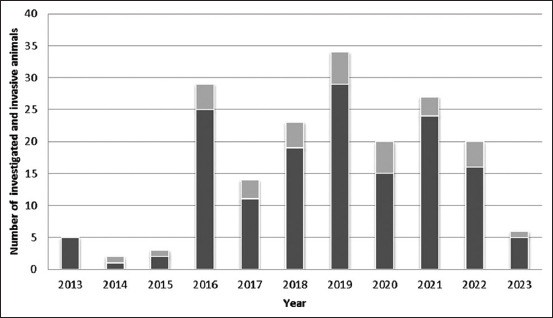
The number of animal carcasses and infected animals examined for trichinellosis infection from October 2013 to February 2023.

Figure-2 shows the results of studies conducted in 2013–2023, including the number of animals studied and the invasive level. In 2016, 25 wild wolves were captured in the Akmola, Atyrau, and Karaganda regions which were investigated, of which four were infected. In 2017–2019, a total of 59 animals (wolves, wild boars, foxes, and corsacs) were captured in five regions of which 12 were infected. In 2020–2023, 60 animals (wild boars, badgers, foxes, and wolves) were studied in five regions, among which 13 were infected.

In total, 155 carcasses of wild carnivorous animals were captured and examined during the research period, of which 98 were from wolves from the Akmola, Karaganda, Ulytau, Kostanay, West Kazakhstan, and Aktobe regions. Among the wolf carcasses, 20 were infected with *Trichinella* larvae and captured in the Karaganda (n = 7), Akmola (n = 1), Ulytau (n = 1), Kostanay (n = 6), West Kazakhstan (n = 4), and Aktobe (n = 1) regions. Among the 26 studied fox carcasses, seven were infected and captured in the Akmola (n = 4), Karaganda (n = 1), and West Kazakhstan (n = 2) regions. None of the 13 studied corsacs from the Karaganda (n = 3) and North Kazakhstan (n = 10) regions were infected with *Trichinella larvae*. Likewise, none of the 12 wild boars from the North Kazakhstan (n = 2), Karaganda (n = 5), West Kazakhstan (n = 4), and Kostanay (n = 1) regions were infected. Badgers were from the Akmola (n = 1), North Kazakhstan (n = 2), and Karaganda (3) regions, of which 66.7% were infected. None of the 13 studied corsacs from the Karaganda (n = 3) and North Kazakhstan (n = 10) regions were infected with *Trichinella* larvae (Table-1).

**Table-1:** Number of investigated and invasive animals per each region.

Region	Badger	Wolf	Fox	Corsac	Hog
Akmolinskaya	1/1	16/1	14/4		
Northern Kazakhstan	2/1			10/0	2/0
Karagandinskaya	3/2	27/7	1/1	3/0	5/0
Ulytauskaya		3/1			
Kostanaiskaya		28/6	3/0		1/0
Western Kazakhstan		21/4	8/2		4/0
Aktyubinskaya		3/1			
Total	6/4	98/20	26/7	13/0	12/0

**Figure-3:**
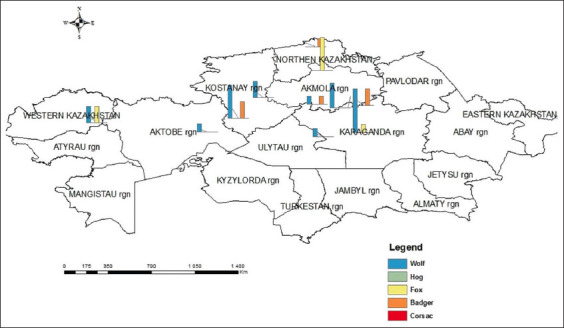
Geographical distribution of trichinellosis among five animal species in February 2023.

Overall, the lowest level of infected animals was recorded in the North Kazakhstan region at 7.1%. Meanwhile, the largest percentages of total infected animals were noted in the Aktobe (33.3%), Karaganda (23.3%), and Kostanay (21.4%) regions, followed by the Akmola (19.3%) and West Kazakhstan (18.2%) regions. The total invasion rate in wolves, foxes, badgers, and wild boars and corsacs were 20.4%, 27%, 66.7%, and 0%, respectively. Figure-3 shows the geographical distribution of infected animals, updated in February 2023.

The level of human infection with the number of infected animals cannot be compared as human infection is spontaneous and unrelated to the number of infected animals. In 2010 and 2011, no infestation was registered among the human population. In 2012–2017, one to three cases of trichinellosis were detected in the East Kazakhstan, Karaganda, and Kostanay regions. In 2018, five cases of human trichinellosis were reported in the Kostanay region. The infection source was the consumption of badger meat without veterinary and sanitary examination. In 2019–2023, no trichinellosis was reported in humans. Since 2004, humans in the West Kazakhstan, Atyrau, Mangystau, and Aktobe regions have been protected against this disease. However, animals had the highest infection rate in the Aktobe region [19].

These results and the recent small outbreaks indicate that public awareness of the risks associated with consuming raw or poorly cooked wild animal meat in Kazakhstan should be increased to improve consumer protection in the region.

### Invasion intensity in foxes, wolves, and badgers

*Trichinella* larvae were counted in the carcasses of foxes, wolves, and badgers to determine the II level. The average weight of three invaded fox carcasses and the average number of larvae of the causative agent of trichinellosis isolated from muscle tissue were calculated (Table-2).

**Table-2:** Intensity of *Trichinella* spp. larvae invasion in carcasses of common foxes.

Animal carcass mass (muscle and bone tissue) (g)	Isolated *Trichinella* larvae (number of specimens)	The II, the number of larvae in 1 g of muscle tissue	

		In the calf muscle group	In the muscles of the animal
3473	13842	11	7
3142	9414	8	4
3890	8476	5	3
Mean value			
3502 ± 216.4	10577 ± 1654.6	8 ± 1.7	4.7 ± 1.2

Abundance index (AI) = 503.6. II=Invasion intensity

**Figure-4:**
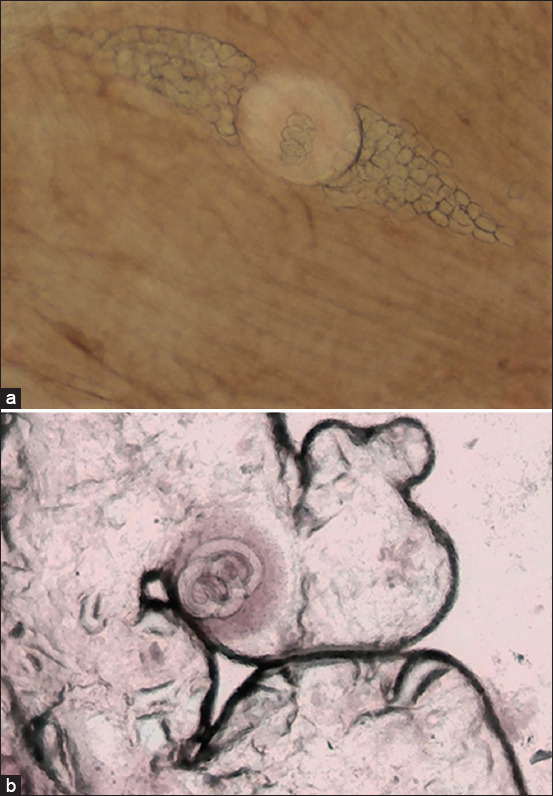
*Trichinella* larvae in the diaphragm (a) and tongue (b) of a fox.

The average number of *Trichinella* larvae in the carcass of a fox weighing 3.7 kg was 10577 ± 1654.6 copies; thus, the average intensity of invasion of one animal is 4.7 *Trichinella larvae* in 1 g of muscle. Simultaneously, the AI of *Trichinella* in this species in the Akmola and Karaganda regions was 503.6 nematode larvae. Moreover, encapsulated larvae were found in the tissues of the diaphragm (Figure-4a) and tongue (Figure-4b) of foxes (Figure-4). The larval distribution in the muscles of foxes is consistent with the literature. The diaphragm, forearm muscles, and tongue are the target sites for *Trichinella* [8]. In Finland, red foxes are critical animal reservoirs for *T. spiralis* and forest *Trichinella* spp. *Trichinella nativa* was the most common species (74%) among wild animals [20].

**Figure-5:**
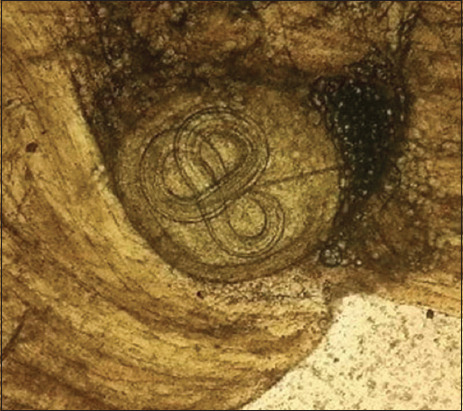
*Trichinella* larvae in a badger diaphragm.

The II was 34.3% per 1 g of muscle in the badger carcasses. The average AI was 164.2 pcs., 10.8 larvae were found in the muscles of the animal (Table-3 and Figure-5). Bears, badgers, and wild boars are the main reservoirs of *Trichinella* invasion in the Tyumen region of Russia. A total of 17 trichinellosis-positive samples were detected during the observation period, of which 58.8% belonged to the badger (*Meles meles*), and the average long-term extent of badger invasion was 18.2%. The primary causative agent of trichinellosis in animals in the Tyumen region is *T. spiralis*, whose dominance index was 94.1% [21]. The II in infected badgers was 14.3%. The largest number of *Trichinella* larvae in the badger is concentrated in the head muscles, and there are no significant differences between body parts [22].

**Table-3:** Intensity of *Trichinella* spp. larvae invasion larvae in badger carcasses.

Animal carcass mass (muscle and bone tissue) (g)	Isolated *Trichinella* larvae (number of specimens)	The II, the number of larvae in 1 g of muscle tissue	

		in the calf muscle group	in the muscles of the animal
14256	11389	38	11
13472	10934	24	8
14095	11621	41	13,5
Mean value			
13941 ± 7033	11314 ± 1741.2	34.3 ± 10.4	10.8 ± 1.80

Abundance index (AI) = 164.2. II=Invasion intensity

**Figure-6:**
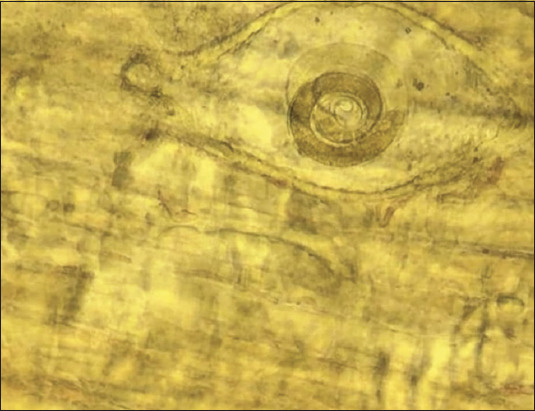
*Trichinella* larvae in the femoral muscle of a wolf.

In the wolf carcasses, the II was almost 2 times higher than in the fox carcasses, both in the calf muscles and, on average, in the muscles of animals (Table-4 and Figure-6). The AI in the muscles of wolves was 289.7 pcs., and 60.6 *Trichinella* larvae were found in the calf muscle of wolves. An average of 4.5 larvae/g was found in the tibial muscle, masticatory muscles, and tongue of the Apennine wolf in Italy. Molecular identification of the larvae showed a mixed infection of *T. britovi* and *T. pseudospiralis* [23]. In Latvia, the invasion level of wolves infected with *Trichinella* spp. was 69.7%, whereas the maximum number of larvae registered in one individual was eight [24].

**Table-4:** Intensity of invasion of *Trichinella* spp. larvae in wolf carcasses.

Animal carcass mass (muscle and bone tissue) (g)	Isolated *Trichinella* larvae (number of specimens)	The II, the number of larvae in 1 g of muscle tissue	

		In the calf muscle group	In the muscles of the animal
20108	17120	67	24
19070	15976	52	17
21413	16023	63	19
Mean value			
20197 ± 8440	16373 ± 1819.2	60.6 ± 6.74	20 ± 2.2

Abundance index (AI) = 289.7. II=Invasion intensity

### Polymerase chain reaction identification of *Trichinella*

**Figure-7:**
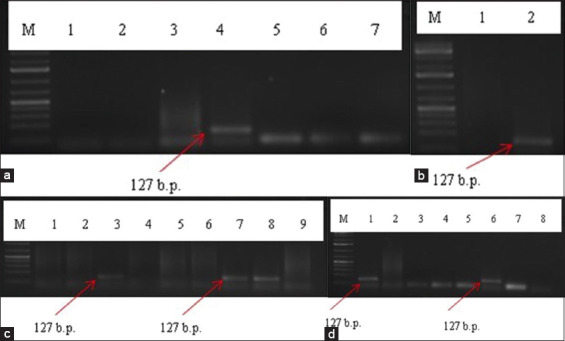
Species identification of *Trichinella* larval DNA samples using polymerase chain reaction. (a) Samples from wild boars in the North Kazakhstan region, 2015. (b) Samples from wolves in the Akmola region, 2016. (c) Samples from wolves in the Karaganda region, 2017. (d) Samples from foxes in the West Kazakhstan region, 2020. An identical amplification product of 127 bp was obtained in all animal species (red arrows). M: Marker (1 kbp); 1–9: Test samples.

More than 30,283 *Trichinella* copies were collected and subjected to detailed molecular genetic analysis to identify the species. A multiplex PCR was conducted using five specific species primers that differentiated all known *Trichinella* genotypes. The DNA fragment sizes of all 155 *Trichinella* samples obtained using multiplex PCR were 127 bp and, thus, were identified as *T. nativa* (Figure-7).

According to the literature in Serbia, *T. britovi* infection was detected in 85% of red foxes and 38% of golden jackals, and *T. spiralis* was detected 15% of red foxes and 63% of golden jackals [25]. In Portugal, the larvae of *Trichinella* spp. were found in one (2.1%) of the 47 foxes examined. After analyzing the multiplex PCR, *T. britovi* was identified as the infecting species [26].

**Figure-8:**
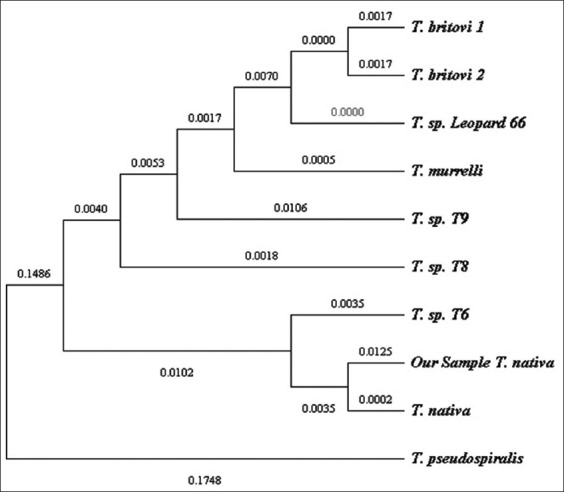
Phylogenetic analysis of the 5S rRNA gene of *Trichinella* spp.

The species affiliation was confirmed by aligning the sequences of the 5S parasite gene isolated from the wolf, which was 577 bp long (Figure-8). Alignment was performed using the sequences of *T. spiralis*, *T. murelli*, *T. britovi*, and *T. pseudospiralis*. The differences in the nucleotide sequence between *T. native* and *T. spiralis* T9 were 21 bp and 15–16 bp between *T. murelli* and *T. britovi*. When aligned with the reference sequence of *T. nativa*, six single-nucleotide substitutions and deletions in our sequence were observed; the deletions were identical in 545–546 bp. However, at the 555 and 564 bp positions, a deletion was found in the reference sequence, which was absent in our sequence.

## Discussion

Trichinellosis is one of the predominant anthropozoonoses, having a natural focal character, and is widespread in many geographical regions. This disease has been reported in more than 120 species of wild and domestic animals and predators, rodents, birds of prey, and humans. Trichinellosis occurs in the USA, Canada, Chile, Argentina, Mexico, Romania, Bulgaria, Yugoslavia, Switzerland, Italy, France, Germany, Great Britain, China, Japan, South Korea, Thailand, and other countries [27–29].

In June 2000, 78 people consumed bear meat infected with *T. nativa* in two communities in northern Saskatchewan, Canada. Confirmed cases were more likely to consume dried meat than boiled meat [30]. In October 2008, a trichinellosis outbreak occurred in northern California, where 30 out of 38 participants fell ill at an event where bear meat was served. Morphological and molecular testing of the muscles from the remaining part of the bear meat showed that the bear was infected with *T. murrelli* [31]. A trichinellosis outbreak due to eating sausages made from wild boar meat that had not been tested for *Trichinella* spp. was registered in Poland in December 2020. This outbreak affected eight people. A study of wild boar meat sausages collected during an epidemiological investigation showed many *Trichinella* spp. [32]. In 2001–2021, 1604 trichinellosis cases were described in Southeast Asia, including Cambodia, Laos, Malaysia, Thailand, and Vietnam. Most cases occurred in the northern regions of Laos (n = 672) and Thailand (n = 773). No disease cases have been reported in Myanmar and the Philippines [33]. From January to February 2015, a trichinellosis outbreak occurred in Genoa, Northern Italy. The epidemiological link was traced back to the dinner served at an agrotourism farm when majority of the 52 guests consumed beef steak tartare. The source of infection was not identified [34]. In Romania, European minks were infected with *T. spiralis*, and wolves, European wildcats, Eurasian lynxes, golden jackals, stone martens, and European badgers were infected with *T. britovi*. Both *Trichinella* species have been found in foxes, bears, wild boars, and stoats [35]. Several dozen to hundred cases of human trichinellosis caused by *T. spiralis*, *T. pseudospiralis*, *T. nativa*, and *T. britovi* are registered annually in the Russian Federation. Trichinellosis in the territory of the Russian Federation is found in many wild and domestic animals, including wolves, jackals, foxes, arctic foxes, raccoon dogs, raccoons, martens, and dogs, among others [36].

Consequently, when studying the level of the spread of trichinellosis among wild carnivores, the primary reservoir for *Trichinella* are wolves, foxes, and badgers. However, only *T. native* was identified in all the studied samples. The early diagnosis of *T. nativa* can be based on the level of creatine kinase in the blood of infected animals [37]. Thus, infected animal species can be used as indicators during routine monitoring studies and should be considered when developing measures to prevent and eliminate trichinellosis in natural biocoenosis.

## Conclusion

Trichinellosis was reported in seven regions of the country, and wolves, foxes, and badgers were infected. Among the 155 animals studied, 31 were infected, most of which were wolves (98/20) and foxes (26/7). Four of the six captured badgers were infected; however, due to the 1 total number of animals, it is impossible to conclude that the species is infected. None of the five corsacs and 12 wild boars studied were infected. Interestingly, the Karaganda, Kostanay, West Kazakhstan, and Akmola regions had the largest distribution of wild animals infected with trichinellosis. Overall, this study obtained a significant number of wild animals infected with *Trichinella*. Thus, regular monitoring of and research on infected animals must be conducted within the country and in the border areas.

## Authors’ Contributions

OSA, LAL, and ASS: Drafted the manuscript and collected the samples. SNB, ZAS, SAA, and AMG: Designed the study. AKZ and FSZ: Performed laboratory analyses. All authors have read, reviewed, and approved the final manuscript.
